# Associations Between Frailty and Inflammation, Physical, and Psycho-Social Health in Older Adults: A Systematic Review

**DOI:** 10.3389/fpsyg.2022.805501

**Published:** 2022-03-14

**Authors:** Kristell Pothier, Wassim Gana, Nathalie Bailly, Bertrand Fougère

**Affiliations:** ^1^Département de Psychologie, Université de Tours, Tours, France; ^2^EA2114, Psychologie des Ages de la Vie et Adaptation, Tours, France; ^3^Division of Geriatric Medicine, Tours University Hospital, Tours, France; ^4^Faculté de Médecine, Université de Tours, Tours, France; ^5^EA 7505 Éducation, Éthique, Santé, Tours, France

**Keywords:** frailty, older adults, biomarkers, physical health, psycho-social health

## Abstract

Frailty is a complex geriatric syndrome with multifactorial associated mechanisms that need to be examined more deeply to help reverse the adverse health-related outcomes. Specific inflammatory and physical health markers have been associated with the onset of frailty, but the associations between these factors and psycho-social health outcomes seem less studied. This systematic review aimed to identify, in the same study design, the potential associations between frailty and markers of inflammation, and physical or psycho-social health. A literature search was performed from inception until March 2021 using Medline, Psycinfo, and EMBASE. Three raters evaluated the articles and selected 22 studies, using inclusion and exclusion criteria (*n* = 17,373; 91.6% from community-dwelling samples). Regarding biomarkers, 95% of the included studies showed significant links between inflammation [especially the higher levels of C-reactive protein (CRP) and interleukin-6 (IL-6)], and frailty status. Approximately 86% of the included studies showed strong links between physical health decline (such as lower levels of hemoglobin, presence of comorbidities, or lower physical performance), and frailty status. At most, 13 studies among the 22 included ones evaluated psycho-social variables and mixed results were observed regarding the relationships with frailty. Results are discussed in terms of questioning the medical perception of global health, centering mostly on the physical dimension. Therefore, the development of future research studies involving a more exhaustive view of frailty and global (bio-psycho-social) health is strongly encouraged.

## Introduction

Frailty is commonly defined as a biologic syndrome correlated with the loss of homeostasis and increased vulnerability to stressors (Fried et al., 2001). While other conceptual models have been suggested (Rockwood and Mitnitski, 2007; Panza et al., 2015), Fried’s phenotype represents the most frequently used one to measure frailty (Fried et al., 2001). Fried’s phenotype focuses on a unidimensional physical construct and defines frailty by the presence of at least three of the five following elements: unintentional weight loss, low grip strength, exhaustion, slow gait speed, and low physical activity level (pre-frailty status is defined by the presence of one or two criteria). According to this phenotype, approximately 10% of people over 65 years old and 25–50% of those over 85 years are being frail (Fried et al., 2001). A more recent meta-analysis suggested that community-dwelling older adults were prone to developing frailty, with a pooled incidence rate being 43.4 cases per 1,000 person-years (Ofori-Asenso et al., 2019). This frequent age-related syndrome has an important negative impact on health outcomes as it is commonly associated with an increased risk of incident falls, worsening mobility or disability, hospitalization, and death (Fried et al., 2001). On a positive note, research studies have shown that frailty was a dynamic process, with possible fluctuations between frailty states for individuals (Pollack et al., 2017; Trevisan et al., 2017). The influence of the life trajectories of older adults will influence the emergency and impact of frailty situations, increasing the inter- and intra-individual variability. To better understand frailty mechanisms is then crucial to identify as early as possible relevant modifiable factors and help create efficient and personalized interventions (mostly including physical exercise, but also nutritional and cognitive trainings) to delay or reverse frailty.

Regarding biological mechanisms, the development and progression of frailty have often been associated with a systemic inflammatory state. The recent systematic review and meta-analysis from Soysal et al. (2016) compared the inflammatory profile of frail and pre-frail with non-frail older subjects (*n* = 23,910, mean age of 75.2 ± 6.1 years). Results of cross-sectional studies highlighted specific biomarkers associated with frailty: frail and pre-frail individuals had significantly higher levels of pro-inflammatory cytokines, such as C-reactive protein (CRP), interleukin-6 (IL-6), and tumor necrosis factor-alpha (TNF-α), as well as the higher levels of fibrinogen and white blood cells (WBC) counts vs. non-frail participants. Specific pathways leading to frailty and involving these pro-inflammatory biomarkers have been studied (e.g., metabolically active fat depots, activation of common molecular pathways in several interactive physiological systems, and inflammatory cascades; as shown in ref. Walston et al., 2006) indicating that inflammation seems to be an important pathophysiological change associated with frailty.

Inflammatory biomarkers could also play an indirect role in the presence of physical declines associated with frailty (more specifically with physical frailty according to Fried’s phenotype). Among these age-related declines, sarcopenia (i.e., reduced muscle mass associated with limited mobility; Morley et al., 2011) has been considered as an important parameter of physical frailty. As noted by Landi et al. (2015), sarcopenia might be considered both as the biological substrate for the development of physical frailty (particularly low grip strength, slow gait speed, and low physical activity level), and the physiopathologic pathway which could result in future adverse health outcomes (mobility disability, falls, loss of independence, …). Moreover, previous research studies have shown that sarcopenia was characterized by the increased levels of pro-inflammatory cytokines, such as TNF-α, IL-6, or CRP (Vatic et al., 2020), which could directly or indirectly speed up frailty. Inversely, the age-related significant rise of inflammatory markers (also known as “*inflammaging*,” as shown in ref. Franceschi et al., 2000) could predispose older individuals to sarcopenia (Liguori et al., 2018) and frailty.

The associations between physical frailty, inflammation, and physical health, while being not fully understood yet, are well-documented in the aging literature. Nevertheless, frailty is more than just physical declines. It represents a multidimensional syndrome involving physical, functional, cognitive, and psychosocial interactions (e.g., cumulative deficit model by Rockwood and Mitnitski, 2007). Psycho-social health markers do need to be taken into greater account in studies examining the markers of (physical) frailty. In their review, Zaslavsky et al. (2013) mentioned various psycho-social indicators associated with frailty, such as cognition, depressive symptoms, or lifestyle factors (such as low-educational level and poor socioeconomic conditions). Frailty and specific psycho-social health outcomes (such as cognitive decline, as shown in ref. de Morais Fabrício et al., 2020 or depressive symptoms, as shown in ref. Zalli et al., 2016) do share common risk factors, such as increased pro-inflammatory cytokines but future studies would be needed to specifically evaluate the potential associations between inflammatory markers and various psycho-social outcomes in frail older adults. This is particularly important considering the protective role of some psychosocial factors against the onset and the worsening of frailty among older adults. For example, a recent review (Sardella et al., 2020) showed that education, occupation, premorbid intelligence quotient, and leisure time activities (as cognitive reserve factors) were able to interact with the frailty status of older adults.

Frailty is a complex geriatric syndrome with multifactorial associated mechanisms that need to be more deeply examined. One possible and innovative avenue of research to better understand the direct and indirect contributions to and of frailty would be to observe the relationships between this syndrome and inflammation/physical health/psycho-social health in the same study design. Therefore, the purpose of this original study was to perform a systematic review on cross-sectional studies about frailty in older adults to identify potential associations with the markers of inflammation, and with physical or psycho-social health.

## Methods

### Study Design

A systematic review was conducted, following Mulrow’s recommendations (Mulrow, 1994), to describe the current state of knowledge regarding the associations between frailty and inflammatory, physical, and psycho-social outcomes to provide recommendations for future research studies.

### Search Strategy

Systematic literature research from inception until March 2021 was conducted using Medline, Psycinfo, and EMBASE with the following search terms: (frail* [MeSH Terms]) AND ((“inflam*”[MeSH Terms] OR “inflam*”[All Fields])) AND (((Health) OR (Health Status) OR (Mental Health))).

### Selection of Studies

Study selection was conducted in two steps. First, three independent authors (KP, WG, and NB) reviewed all titles and abstracts using the following inclusion and exclusion criteria. Studies involving: (1) older adults (population with a mean age >65 years old), (2) a specific measure of frailty, and (3) specific inflammatory biomarkers were included. Duplicates, studies that were not in English or French, and studies that were not cross-sectional (longitudinal, interventional or protocol studies, reviews, book chapters, comments, or editorials) were excluded. Second step involved retrieving the full text of the selected papers, and filtering them for relevance using an additional criterion: to be included, papers must evaluate the potential associations between frailty and either inflammation or physical health or psychosocial health. Finally, the three reviewers discussed the papers and agreed on final inclusion.

### Data Collection

The following information has been extracted from the selected studies: author(s) and year of publication, characteristics of participants (such as, size, mean age, and percentage of men and women), and measures used to characterize frailty, types of inflammatory biomarkers, and physical and/or psycho-social health (data summarized in Supplementary Table 1). Two of the co-authors (WG and KP) screened all the markers used in the included studies to highlight specific inflammatory biomarkers. Other biomarkers having a role in the inflammatory process (such as oxidative stress markers or muscle protein turnover) were then considered as the markers of older adults’ general physical health. The inter-judge procedure (involving the three co-authors WG, NB, and KP) was also performed for all physical and psychosocial health outcomes included in the studies. Mean results on the potential associations between the three outcomes (inflammation, physical health, and psycho-social health) with frailty, and specific relationships among the three outcomes (when data were available) are presented in Supplementary Table 2.

## Results

**FIGURE 1:**
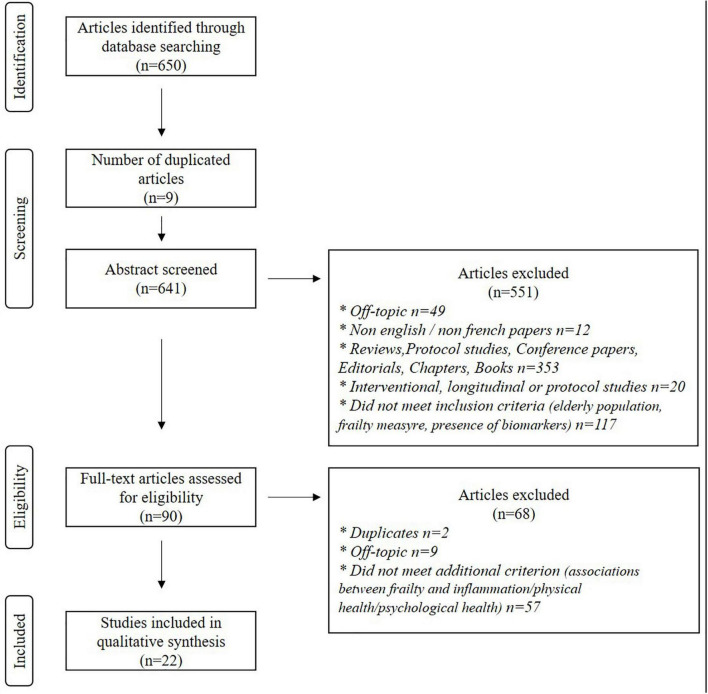
The flowchart of studies.

The flowchart (Figure 1) shows the number of studies identified from databases (*n* = 650). After removing duplicates and screening articles based on abstracts, 90 records remained. The full-text articles reading led to the exclusion of 68 studies (2 duplicates, 9 off-topic, and 57 studies that did not evaluate the potential associations between frailty and inflammation/physical health/psycho-social health). Accordingly, 22 studies met the pre-established criteria and were included in this systematic review.

### Main Characteristics of the Studies Included

Supplementary Table 1 shows the main characteristics (population and measurements) of the studies included.

Thirteen of the 22 included studies (59%) involved a community-dwelling sample. Six studies involved vulnerable older adults, such as followed-up for chronic diseases (Boxer et al., 2008; Wu et al., 2009; Lee Y. P. et al., 2016; Huang et al., 2020), veteran (Van Epps et al., 2016), and socially vulnerable older adults (Nascimento et al., 2018). The three remaining studies involved institutionalized (Fernández-Garrido et al., 2014) and hospitalized (Ma et al., 2018; Yang et al., 2018) older adults.

Included studies represent a total of 17,373 older adults [*n* ranging from 30 (Leng et al., 2002) to 4,735 (Walston et al., 2002)]. The community-dwelling sample contains 15,912 older adults (91.6%), while the total number of vulnerable older adults and inpatients were respectively 854 (4.9%) and 607 (3.5%).

It should be noted that five studies (Blaum et al., 2009; Fried et al., 2009; Chang et al., 2012; Silva et al., 2014; Van Epps et al., 2016) did not specify the mean age of their samples. Taking into account the 17 other studies, the total mean age of the included older adults was 75.34 years [means ranging from 65.5 (Lee W. J. et al., 2016) to 84.9 years (Leng et al., 2002)]. The total mean age of inpatients and institutionalized older adults (79.53, *n* = 3 studies) were higher than the vulnerable older adults one (75.67; *n* = 5 studies), which was also higher than the total mean age of the community-dwelling older adults (74.24; *n* = 9 studies).

Regarding sex ratio, included studies involved a small majority of older women, with a total mean percentage of women of 58.4%. Of important note, five studies (Leng et al., 2007; Blaum et al., 2009; Fried et al., 2009; Chang et al., 2012; Fernández-Garrido et al., 2014) included a 100%-women sample while only one included a 100%-men sample (Lee W. J. et al., 2016). Comparing sex ratio between the different samples, a lower percentage of older women in the studies involving vulnerable older adults (39.3% of women) compared with inpatients (54.6%) and community-dwelling older adults (67.8%) studies was noted.

### Measures

Data regarding measurements are summarized in Supplementary Table 1.

While measures of frailty and inflammation were mandatory for studies to be included in this systematic review (first step of the studies selection), physical and psychosocial health measures were optional (second step of the studies selection). Data are summarized in Supplementary Table 1.

Regarding frailty, 100% of the studies used Fried criteria (weight loss, exhaustion, weakness, slow walking speed, and low physical activity; Fried et al., 2001) to characterize frailty. A large majority of the studies (17/22, 77.3%) divided their sample into three groups: non-frail, pre-frail (one or two criteria), frail (≥three criteria) based on the Fried phenotype of frailty (Fried et al., 2001). Three studies (Leng et al., 2002; Chang et al., 2012; Yang et al., 2018) divided their sample into two groups (non-frail vs. frail older adults) with two of them (Chang et al., 2012; Yang et al., 2018) considering non-frail status as having from 0 to 2 Fried criteria. One study divided the included sample into four frailty groups (sub-dividing the pre-frail group into a low frailty group—older adults having one criterion—and a medium frailty group—two criteria—Boxer et al., 2008). Last, one included study (Fernández-Garrido et al., 2014) chose to specifically consider frailty score as a continuous variable only, and five individually examined the five frailty criteria (Fernández-Garrido et al., 2014; Silva et al., 2014; Ma et al., 2018; Semmarath et al., 2019; Huang et al., 2020).

Regarding inflammation, among the 22 included studies, 15 (68.2%) studies measured Interleukin (IL; mostly IL-6) levels, and/or C-reactive protein (CRP), or high sensitive CRP (hsCRP) levels, 8 (36.4%) studies included a white blood cell (WBC) count, 5 (22.7%) studies examined tumor necrosis factors (TNFs; mostly TNF-α), and 3 studies (13.6%) included measures of hemostatic factors (fibrinogen, Factor VII, Factor VIII, transferrin, and haptoglobin). More sporadically, vascular adhesion protein-1 (VAP-1; Huang et al., 2020) and erythropoietin (EPO; Silva et al., 2014) were also measured. Of important note, 3 studies also calculated an inflammation index score (Chang et al., 2012; Van Epps et al., 2016; Ma et al., 2018).

The totality of the included studies evaluated physical health. The following measurements were considered falling under physical health: biochemical measurement (*n* = 17 studies, 77.3%), anthropometric measures (*n* = 13, 59%), comorbidities (*n* = 14, 63.7%), smoking and alcohol status (*n* = 10, 45.5%), medications (*n* = 6, 27.3%), physical performance (physical activity, grip strength, gait speed, energy level, and fine motor speed; *n* = 4, 18.2%), blood pressure (*n* = 2; Lee Y. P. et al., 2016; Ma et al., 2018), past medical history (*n* = 2; Hsieh et al., 2018; Semmarath et al., 2019), nutritional status (*n* = 1; Hsieh et al., 2018), falls or the risk of falls (*n* = 2; Fernández-Garrido et al., 2014; Darvin et al., 2014), overnight hospital admissions (*n* = 1; Zhu et al., 2016).

In the 22 included studies, 13 (59%) studies involved psycho-social variables. Among them, 10 (77%) included lifestyle characteristics (years of education, marital status, and capital income) of older adults, 7 (53.8%) effective measures, and 6 (46.1%) measured cognition (MMSE, memory loss, and subjective cognitive decline). Behavioral disorders and autonomy (i.e., functional status) were also evaluated in one study (Fernández-Garrido et al., 2014).

### Significant Associations Between Frailty and the Three Specific Outcomes

Supplementary Table 2 shows all the found associations between frailty and specific outcomes included in this systematic review.

#### Associations Between Frailty and Inflammatory Biomarkers

Only one study did not find a significant relationship between frailty and inflammation (Lee Y. P. et al., 2016). Among the 21 left studies, 13 of them (62%) found significant links between frailty and the totality of the used inflammatory biomarkers; the remaining studies showed links between frailty and specific inflammatory biomarkers. IL-6 and CRP levels were significantly and positively associated with frailty in, respectively, 15 (100%) and 12 (80%) of the studies using these cytokines biomarkers. Four studies over the 8 including WBC levels significantly and positively linked this inflammatory measure with frailty (50%). TNFs were significantly and positively associated with frailty in 2 of the 5 studies (40%) using these biomarkers. Two studies over the three measuring hemostatic factors found a significant and positive association with frailty (66%). The studies using more sporadic inflammatory measures found mixed results: while VAP-1 was significantly and positively associated with frailty in Huang et al. (2020), EPO did not correlate with frailty in Silva et al. (2014). All the studies using an inflammation index score showed significant and positive relationships with frailty.

A large majority of studies comparing an inflammation between frailty groups (*n* = 12/13) showed that frail individuals had significantly higher levels of pro-inflammatory cytokines (CRP, IL-6, and TNF), WBC, hemostatic factors, and VAP-1 levels, compared with non-frail, and, to a less extent, pre-frail older adults.

Nine studies evaluated the potential links between frailty and inflammation with correlations and regressions analyses. All of them showed significant positive correlations between specific biomarkers and a higher frailty phenotype score (Boxer et al., 2008; Fernández-Garrido et al., 2014) or and the likelihood of being pre-frail or frail (Leng et al., 2007; Blaum et al., 2009; Wu et al., 2009; Chang et al., 2012; Darvin et al., 2014; Silva et al., 2014; Lee W. J. et al., 2016).

Among the five studies evaluating individual frailty criteria, three of them found direct links between the low grip strength criterion and specific biomarkers (IL-6 and CRP in Semmarath et al., 2019; WBC count in Fernández-Garrido et al., 2014; VAP-1 levels in Huang et al., 2020), and two of them between the exhaustion criterion and specific biomarkers (IL-6 and IL-1ra levels in Silva et al., 2014; VAP-1 levels in Huang et al., 2020). A study also linked the low physical activity level criterion with WBC count (Fernández-Garrido et al., 2014). Finally, one study associated the slow gait speed criterion with higher IL-6 levels (Ma et al., 2018).

#### Associations Between Frailty and Physical Measures

Among the 22 studies evaluating physical health outcomes, 3 of them did not find any significant association with frailty at all (Leng et al., 2007; Fernández-Garrido et al., 2014; Van Epps et al., 2016).

Regarding biochemical measures, over 17 studies including them, 14 reported significant associations with frailty (82.3%). These studies mainly showed that frail older adults had lower levels of red blood cell (*n* = 4), albumin (*n* = 3), vitamin D (*n* = 1), AST and ALT (*n* = 2), urea (*n* = 1), reticulocyte (*n* = 1), intracellular adhesion molecule-1 (*n* = 1), and higher levels of creatinine (*n* = 2), cholesterol (*n* = 2), procalcitonine (*n* = 1), oxidative stress (8-OHdG, dROM, TTL; *n* = 2), zinc alpha2-glycoprotein (*n* = 1), triglyceride (*n* = 1), compared with non-frail older adults. Mixed results were found for hemoglobin but a majority of the studies (4/7) showed lower levels for frail patients, compared with non-frail older adults (one reported higher levels of hemoglobin for frail individuals, the other two did not find significant differences). Among the 13 studies taking into account of the anthropometric measures, 4 reported a significant relationship with frailty (30.8%), with higher BMI values associated with frail status. Twelve of the fourteen studies measuring comorbidities reported a link between the presence of specific diseases (mostly cardiovascular disease, diabetes, hypertension, arthritis, and stroke) and frailty (85.7%). Among the 10 studies measuring smoking and alcohol status, 3 reported significant associations (30%), with more current and former smokers in the frail groups (Saum et al., 2015; Lee W. J. et al., 2016) and more drinkers in non-frail and pre-frail older adults (Saum et al., 2015; Semmarath et al., 2019). Two studies over the six measuring number of medications (33.3%) reported a significant relationship with frailty (statins and thiazolidinediones; Lee Y. P. et al., 2016; Huang et al., 2020). Over the four studies evaluating specific physical measures, all of them (100%) reported a significant association with frailty, with a global lower physical performance associated with frailty status (Fried et al., 2009; Darvin et al., 2014; Ma et al., 2018; Nascimento et al., 2018). More sporadically, the few studies evaluating past medical history (*n* = 2), nutritional status (*n* = 1), and overnight hospital admissions (*n* = 1), all showed a significant relationship with frailty.

Regarding analyses performed on individual frailty criteria (*n* = 5 studies), only one reported a significant relationship with physical health measures (Silva et al., 2014). Higher values of Red blood cell Distribution Width (RDW) (measuring the variation in red blood cell size) were associated with the presence of exhaustion and slow gait speed criteria while the lower levels of reticulocyte increasing the change of being positive for the low physical activity criterion.

#### Associations Between Frailty and Psycho-Social Measures

Among the 13 studies measuring psycho-social variables, 3 of them did not find any significant association with frailty at all (Darvin et al., 2014; Fernández-Garrido et al., 2014; Ma et al., 2018); 6 studies linked all their used measures with frailty (Leng et al., 2007; Wu et al., 2009; Chang et al., 2012; Lee W. J. et al., 2016; Hsieh et al., 2018; Yang et al., 2018), and 4 studies found partial links (associations between frailty and specific psycho-social measures; Blaum et al., 2009; Zhu et al., 2016; Nascimento et al., 2018; Semmarath et al., 2019).

Regarding lifestyle characteristics, 5 of the 10 studies including these measures (50%) found significant relationships with frailty. Results showed that frail and pre-frail participants were less educated than non-frail older adults, but mixed results were found considering marital status (only one over the five studies measuring this variable found that frail older adults were more likely to be unmarried compared with non-frail individuals; Yang et al., 2018). Regarding affective status, three of the seven studies including this variable (43%) found significant differences in the prevalence of depressive symptoms between frailty groups (Wu et al., 2009; Chang et al., 2012; Nascimento et al., 2018, with more depressive symptoms in frail compared with non-frail older adults). Among the six studies evaluating the cognitive status, three of them (50%) found significant differences between frailty groups, with frail participants having lower cognitive scores (Chang et al., 2012; Hsieh et al., 2018; Semmarath et al., 2019) or higher cognitive subjective decline (Hsieh et al., 2018) than non-frail older adults.

#### Total Number of Associations Between Frailty and Specific Outcomes

Among the 22 included studies, 9 of them (41%) found significant associations between frailty status and the 3 inflammatory/physical/psycho-social measures. Ten studies (45.5%) found a double-association with nine of them significantly linked frailty with inflammation and physical health measures while only one linked frailty with inflammation and psycho-social health measures (Leng et al., 2007). Finally, three studies found a single significant association, with two studies showing a relationship between frailty and inflammation (Fernández-Garrido et al., 2014; Van Epps et al., 2016) while the other one found a link between frailty and physical health markers (Lee Y. P. et al., 2016). Of important note, these three studies included samples composed of vulnerable outpatients or institutionalized older adults.

### Relationships Between Inflammation, Physical, and Psycho-Social Health Outcomes

When available, data regarding relevant associations between specific outcomes included in this systematic review have been summarized in Supplementary Table 2.

Among the 22 included studies, 7 (31.8%) studies reported results on relationships between inflammation, physical health, or psycho-social health (associated or not with the relationship with frailty). Independency between specific factors was found in three studies (subjective cognitive decline and inflammation in Hsieh et al., 2018; ICAM-1 and IL-6 in Lee W. J. et al., 2016; and WBC and all geriatric assessments in Fernández-Garrido et al., 2014). Significant relationships were found between inflammatory and physical health markers in three studies (Boxer et al., 2008: lower hsCRP and IL-6 levels correlated with intact parathyroid hormone levels; Saum et al., 2015: positive correlations between markers of oxidative stress; Zhu et al., 2016: the higher levels of hsCRP associated with overnight hospital admission) while a physical health outcome (HbA1c) was negatively correlated with life-style characteristics (educational level) in one study (Blaum et al., 2009).

## Discussion

This original systematic review of the aging literature examined the potential associations between frailty states and inflammatory, physical, and psycho-social markers of health.

The population included in this review is largely composed of community-dwelling older adults (over 90%). The main focus of cross-sectional studies on this specific elderly population is not surprising: due to its dynamic process, the frailty syndrome can be more easily reversed if interventions target older adults before major clinical events (such as emergency room admissions or hospitalizations; as shown in ref. Vellas et al., 2013). To better understand frailty mechanisms in this key population is thus of great interest. Nevertheless, more studies involving frail older adults with multi-system impairments would also be helpful to further propose the best treatment, related to each independent prognosis’ condition (i.e., frailty syndrome vs. other specific comorbidities; Fried et al., 2004; Hoogendijk et al., 2019).

While, decades ago, the World Health Organization (WHO) defined health as “a state of complete physical, mental, and social wellbeing and not merely the absence of disease or infirmity” (Shimkin, 1946), aging research and clinical studies still perceive health from a medical point of view, centering mostly on the physical dimension of health. This medical view is represented in included measures of the main outcomes of this systematic review. While different tools exist to clinically measure frailty in older adults (depending on frailty approaches; e.g., Rockwood and Mitnitski, 2007; Panza et al., 2015), all the included studies have used Fried’s criteria, a fast and easy-to-use frailty measure, frequently employed in medical care. Regarding inflammatory biomarkers, a large majority of the included studies, whatever the sample of older adults, measured pro-inflammatory cytokines (CRP, IL-6, and TNF) and WBC, also frequently quantified in medical units. Moreover, the totality of the included studies measured physical health through various domains (e.g., biochemical measurement, anthropometric measures, comorbidities, and physical performance), while a lower number of studies (59%) included a psycho-social health assessment.

Main results on the associations between *frailty and inflammation* highlighted the central role of specific cytokines in this geriatric syndrome. The totality of included studies measuring IL-6 level and 80% of the studies involving CRP showed that frail older adults had higher levels of both of these biomarkers compared with non-frail participants. These results were confirmed in studies with regression analyses, even if the different methods involved in the odd-ratios (*OR*s) calculations made the comparisons more complex to do (as shown in refs. Leng et al., 2007 vs. Lee W. J. et al., 2016). These overall results regarding specific inflammatory biomarkers, also found in a recent meta-analysis (Marcos-Pérez et al., 2020), confirmed the existing literature on the inflamm-aging paradigm in frailty older adults (as shown in ref. Vatic et al., 2020 for a review) and support the role of age-related chronic inflammation in frailty development. Results on *physical health measures* showed important relationships with frailty. Regarding biochemical measurement, results mostly showed that, compared with non-frail older adults, frail individuals had lower levels of red blood cells, especially hemoglobin (more than 50% of the studies reported a significantly negative relationship with frailty). Of important note, one study analyzing individual Fried’s criteria reported a significant relationship between red blood cells and two frailty criteria (exhaustion and slow gait speed; Silva et al., 2014), and another one reported increasing BMI values associated with increasing hemoglobin levels (Blaum et al., 2009). Taken together, these results could be in line with previous studies linking red blood indices to frailty through sarcopenia (Silva et al., 2014; Vatic et al., 2020), even if more studies would be needed to confirm this hypothesis. This review confirmed the strong links between comorbidities and frailty: the older adults suffer from specific diseases, the more they are at risk of being frail. The inter-relationships between comorbidity and frailty has often been reported (as shown in Ref. Gobbens et al., 2010) even if research studies still lack to determine whether comorbidities act as a cause or as a consequence of adverse outcomes related to frailty. Few studies measured physical performance (4/17), but all of them reported significant links with frailty. These results are not surprising considering how included studies defined and measured frailty in older adults. Fried’s criteria, and especially low grip strength, slow gait speed, and low physical activity level, will automatically imply a significantly reduced physical performance in frail individuals. Finally, in this review focusing on frailty and inflammatory biomarkers (two medical concepts), *psycho-social health* has been under-measured comparing with a physical assessment and studies produced mixed results. Less than 50% of the included studies found significant associations among educational level, marital status, cognition, or depression and frailty status. It could be hypothesized that frailty, and especially physical frailty, impacts to a less extent psycho-social health, compared with physical health or inflammation. Nevertheless, previous reviews of the literature have linked frailty to psycho-social measures in older adults. For instance, poor mental health (and especially the presence of depressive symptoms, usually measured with Center for Epidemiologic Studies-Depression (CES-D) or Geriatric Depression Scale (GDS) scales) is frequent in frail older adults (as shown in ref. Buigues et al., 2015, for a systematic review). It has been hypothesized that physiological dysregulation associated with frailty (generating low-grade inflammation, for example) could predispose or precipitate depression in aging (as shown in ref. Buigues et al., 2015). Regarding cognitive status, a recent meta-analysis did show that frail older adults were at a higher risk of incident cognitive disorders (measured through neuropsychological testing) than non-frail elders (pooled *OR* = 1.80, 95% *CI* = 1.11–2.92; *p* = 0.02; Borges et al., 2019). Mixed findings observed in this systematic review could be the result of the simple tools used in lots of included studies to measure complex psycho-social concepts. For instance, the MMSE test is frequently used in frailty studies to measure cognitive impairment. Nevertheless, when studying cognition in community-dwelling samples, the use of more complex tests, evaluating executive functions for instance (one of the first cognitive functions to be early affected in normal aging; Amieva et al., 2003) could provide interesting avenues of research in the field of frailty. This rationale is also true for mental health, a complex concept involving both environmental (e.g., social support) and personal (e.g., self-efficacy) factors, and not just the absence of depressive symptoms (which are assessed here in a variety of ways). Future research studies analyzing the various causes and effects of frailty should also include precise measures of psychological variables to not miss out on an important part of older adults’ health.

In addition, this systematic review raised some important remarks in the research field of frailty. First, the importance of intermediate states could be underestimated, at least, by few frailty studies exploring inflammatory biomarkers. For instance, two included studies considered participants as being non-frail when having from 0 to 2 Fried’s criteria (Chang et al., 2012; Yang et al., 2018) while pre-frail status has been associated with specific patterns of results (on some measures, pre-frail older adults acted similarly as non-frail individuals whereas, on others, they acted like frail individuals). Moreover, on some specific physical health measures, the pre-frail group was the only one showing signs of poorer health [higher hypercholesterolemia levels or lower grip strength (Darvin et al., 2014), or higher hyperlipidemia (Huang et al., 2020), in pre-frail individuals compared with the two other groups]. Pre-frail individuals could then represent a target population, between prevention and intervention, to focus on delay or avoid adverse health outcomes related to frailty. Second, this systematic review highlighted the fact that frailty studies, when exploring inflammatory biomarkers, still lack a holistic approach of health. Future studies would be needed to specifically explore psycho-social health and its relationship with inflammation and frailty. The use of other conceptual models on frailty, such as the accumulation of age-related deficits, proposed by Rockwood and Mitnitski (2007), would be interesting to explore deeper frailty and inflammation impact on physical and psycho-social health in future studies. The underlying idea would be to further investigate the bidirectional links between psychological and physical health (e.g., is subjective well-being a cause or effect of physical health? As shown in ref. Gana et al., 2013), particularly in frail older adults, and using inflammatory biomarkers as potential mediators.

While innovative and exploratory, this systematic review contains some limitations worth pointing out. First, the associations between frailty and inflammation, physical health, and psycho-social health, were only observed in cross-sectional studies, limiting the findings impact. Previous longitudinal studies have shown strong links among inflammatory markers (Gale et al., 2013), physical decline (Gobbens and van Assen, 2014), and psycho-social (specifically, cognitive impairment; Samper-Ternent et al., 2008), and the onset of frailty but a recent meta-analysis on inflammation and frailty (Soysal et al., 2016) pointed out methodological bias (paucity of data, over-adjustment of the analyses due to various baseline potential confounders in the included studies, …). Therefore, this systematic review voluntarily focused on cross-sectional studies only. Second, data regarding specific multiple associations among frailty, inflammation, physical health, and psycho-sociological health were hard to retrieve in the studies because the studies analyses mainly focused on frailty status differences (group comparisons or multivariate regressions). This made the comparisons between studies harder but inclusion criteria used in this systematic review were partly responsible for it. Third, the use of unspecific MeSH Terms regarding frailty and inflammation may have led to miss few specific references. However, this broad search strategy was voluntarily employed to retrieve as many as possible medical or physiological studies, and study how they included any physical or psycho-social health evaluation.

This systematic review is the first one, to the best of our knowledge, to explore, in the same study design, the relationships between frailty and three markers of health (inflammation, physical health, and psycho-social health). While results have mostly confirmed existing literature regarding the strong links between frailty status and inflammation or physical health decline, studies evaluating psycho-social health of frail older adults still lack when inflammatory biomarkers and Fried’s criteria (two medical concepts) are involved. Therefore, the development of future research studies is strongly encouraged: (1) to deeper explore the causal relationships between all these markers (top-down vs. bottom-up approaches), and (2) with a more exhaustive view of frailty and global health.

## Data Availability Statement

The original contributions presented in the study are included in the article/Supplementary Material, further inquiries can be directed to the corresponding author.

## Author Contributions

All authors listed have made a substantial, direct, and intellectual contribution to the work, and approved it for publication.

## Conflict of Interest

The authors declare that the research was conducted in the absence of any commercial or financial relationships that could be construed as a potential conflict of interest.

## Publisher’s Note

All claims expressed in this article are solely those of the authors and do not necessarily represent those of their affiliated organizations, or those of the publisher, the editors and the reviewers. Any product that may be evaluated in this article, or claim that may be made by its manufacturer, is not guaranteed or endorsed by the publisher.
